# Malignancy-Associated Secondary Hemophagocytic Lymphohistiocytosis Mimicking an Infection: A Case Report and Review of the Literature

**DOI:** 10.7759/cureus.56738

**Published:** 2024-03-22

**Authors:** Meenakshi Gopalakrishnan, Arunalini Ramanathan, Dhaarani Jayaraman, Sri Gayathri Shanmugam, Julius Xavier Scott

**Affiliations:** 1 Pediatrics, Sri Ramachandra Institute of Higher Education and Research (SRIHER), Chennai, IND; 2 Pediatric Hematology and Oncology, Sri Ramachandra Institute of Higher Education and Research (SRIHER), Chennai, IND; 3 Pathology, Sri Ramachandra Institute of Higher Education and Research (SRIHER), Chennai, IND; 4 Pediatric Haematology and Oncology, Sri Ramachandra Institute of Higher Education and Research (SRIHER), Chennai, IND

**Keywords:** lymphadenopathy, hematopoietic stem cell transplant, crizotinib, hemophagocytic lympho histiocytosis (hlh), non-hodgkin’s lymphoma, pediatric nhl, anaplastic large cell lymphoma

## Abstract

Hemophagocytic lymphohistiocytosis (HLH) is a rare, life-threatening hematological disorder of immune dysregulation associated with significant challenges in diagnosis and management. Described as primary HLH secondary to genetic defects or more commonly secondary to infections, it can also occur secondary to malignancy, i.e., malignancy-associated hemophagocytic lymphohistiocytosis (M-HLH).

A five-year-old male child presented with left cervical adenopathy and a high-spiking fever for two weeks. He had pallor, anasarca, multiple enlarged and matted cervical lymph nodes, respiratory distress, and hepatomegaly. He had continuous high-grade fever spikes (maximum 105 °F), not touching baseline despite broad-spectrum antibiotics. The CBC revealed anemia with thrombocytopenia. Liver function tests showed mild transaminitis and hypoalbuminemia. The HLH workup showed elevated ferritin, low fibrinogen, and elevated triglycerides. Lymph node biopsy showed intermediate to large atypical monomorphic lymphocyte cells with ALK, CD30, CD5, CD3, CD45, and BCL-2 (weak positive) positivity and Ki-67-95%, suggestive of anaplastic large cell lymphoma (ALCL). The bone marrow aspiration showed reactive marrow with hemophagocytosis.

The patient was started on dexamethasone and chemotherapy per the Children's Oncology Group's (COG) ALCL protocol. He showed remarkable clinical improvement and went into remission after the induction phase. Malignancy associated with HLH can mimic infection, as in our patient with high-spiking fever, consolidation, and mediastinal adenopathy. A high index of suspicion is necessary to arrive at an appropriate, early diagnosis, and workup for malignancy is to be considered when an infectious etiology is not identified after thorough evaluation.

## Introduction

Hemophagocytic lymphohistiocytosis (HLH) in children is a disorder of immune dysregulation characterized by persistent fever, organomegaly, cytopenias, and hyperferritinemia. It can be primary, secondary to genetic mutations, or secondary to infections, rheumatological conditions, or, rarely, malignant. Hemophagocytic lymphohistiocytosis with malignancy can occur in two forms: infection-associated secondary HLH in view of the underlying immunocompromised status, which can be seen with viruses as triggers, including cytomegalovirus (CMV) or Epstein-Barr virus (EBV); or malignancy-associated HLH (M-HLH), which is usually present at the time of initial diagnosis [1].

Malignancy-associated secondary HLH has been described in various leukemias and lymphomas, especially in T-cell and NK-cell lymphomas. The M-HLH has been described in 1.9% to 8.4% of patients, as per various literature [2]. Here, we report the case of a young boy who had florid M-HLH with the diagnosis of anaplastic large cell lymphoma (ALCL), mimicking an infection. This article was previously presented as a meeting abstract at the Tamil Nadu and Pediatric Oncologist Society (TAMPOS) Conference in Madurai, TN, India, on January 29, 2023.

## Case presentation

The patient was a five-year-old male who presented with left-sided cervical lymphadenopathy and a high-grade fever for two weeks. There was no history of significant weight loss, joint pain or swelling, bleeding, or a history of native medicine intake. He had pallor, anasarca, and multiple enlarged and matted cervical lymph nodes. Systemic examination revealed tachypnea, intercostal and subcostal retractions, and decreased air entry in the right infra-axillary and infra-scapular areas. He had bilateral occasional crepitations and bronchial breath sounds. The abdomen was distended, and he had hepatomegaly.

**Figure 1:**
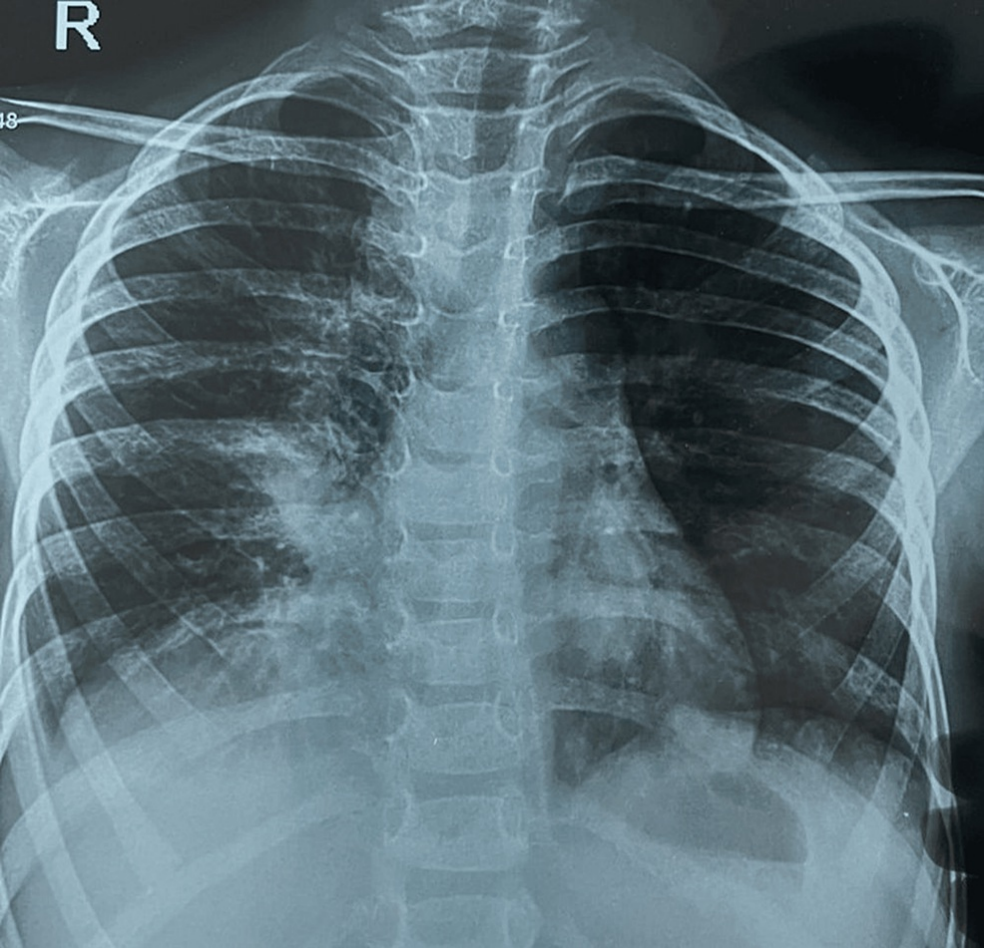
Chest X-ray showing the right middle-lobe consolidation with cardiac, which is silhouetted

The CBC revealed hemoglobin of 7.9 g/dl, WBC of 12,970 cells/mm3, platelets of 58,000 cells/mm3, and the peripheral smear showed microcytic hypochromic anemia with thrombocytopenia. The liver function test (LFT) showed hypoalbuminemia, mild transaminitis, and normal bilirubin. Renal functions and tumor lysis parameters were normal (Table 1). The chest X-ray showed right middle lobe consolidation (Figure 1). The patient was started on broad-spectrum antibiotics after cultures, which were later found to be sterile.

**Table 1:** Salient laboratory features at diagnosis MCV: Mean corpuscular volume, MCH: Mean corpuscular hemoglobin, MCHC: Mean corpuscular HB concentration, CRP: C-reactive protein, ESR: Erythrocyte sedimentation rate, LDH: Lactate dehydrogenase, SGPT: Serum glutamic pyruvic transaminase

Investigations	Values
Hemoglobin	7.9 g/dl
Mean corpuscular volume (MCV), mean corpuscular hemoglobin (MCH), mean corpuscular HB concentration (MCHC)	75.4 fl, 22.2 pg, 30.2g/dl
Total leucocyte count and differentials	12970 cells/mm3 (P 30%, L 62.7%)
Platelet count	0.58 lakhs/mm3
Peripheral smear	Microcytic hypochromic anemia; no blasts
CRP and ESR	1.1 U/L, 65mm/hr
LDH and uric acid	870 U/L, 5.6mg/dl
Liver function tests	SGPT 55U/L, albumin 1.8g/dl, globulin 3.1 g/dl, bilirubin 1.4/ 0.6 mg/dl (total/indirect)
Serum creatinine	0.4 mg/dl

In view of persistent high grade fever spikes (max 105 ° F), not touching baseline despite antibiotics, anasarca, cytopenias, hypoalbuminemia, transaminitis with cervical lymphadenopathy, evaluation for infections, autoimmune causes and HLH was done. Hemophagocytic lymphohistiocytosis workup showed elevated ferritin (5432ng/ml), low fibrinogen (130g/dl) and hypertriglyceridemia (311mg/dl).

**Figure 2:**
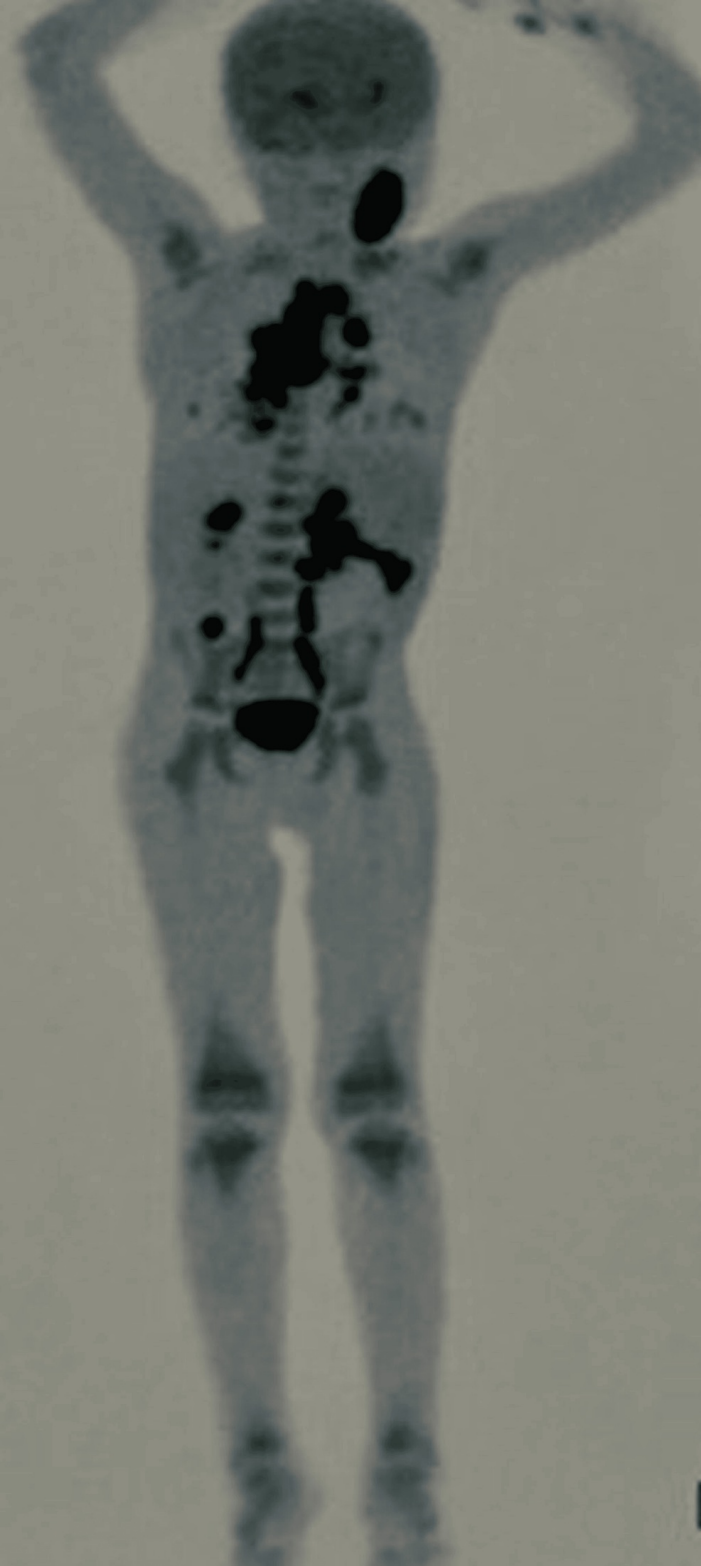
Whole body PET-CT showing conglomerate left cervical nodes, mediastinal and para-aortic lymphadenopathy

The PET-CT showed fluoro deoxy glucose (FDG) avid conglomerate lymph nodes in left cervical levels 2, 3, and 4 with central necrosis in the left supra clavicular, right upper paratracheal, pre-vascular, right lower paratracheal, subcarinal, right hilar, left hilar, conglomerate celiac axis, para-aortic, and pelvic lymph nodes. Right middle lobe lung collapse/ consolidation had bilateral mild to moderate pleural effusion (right>left) with no FDG avid lesions in the liver, spleen an,d bone marrow (Figure 2).

**Figure 3:**
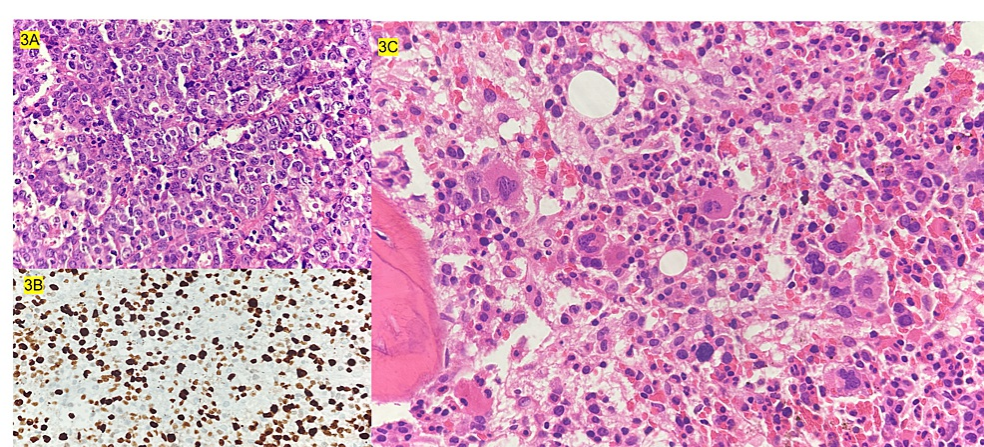
Histopathology images 3A: Sheets of atypical monomorphic cells with high mitotic index; 3B: Diffuse ALK positivity in tumour cells revealed by immunohistochemistry; 3C: Bone marrow aspirate morphology showing increase in hemophagocytosis

Lymph node biopsy showed intermediate to large atypical monomorphic lymphocyte cells with tiny numerous tingible body macrophages with increased mitosis. The immunohistochemistry was positive for ALK, CD30, CD5, CD3, CD45 and BCL-2 (weak positive), KI-67-95%, suggestive of ALCL. Bone marrow aspiration and biopsy revealed reactive marrow with increase in hemophagocytosis (Figure 3).

Final diagnosis of stage 3 AALCL with M-HLH was made. The patient was treated with appropriate supportive measures and also started on dexamethasone at 10mg/sq.m/day per HLH protocol doses. He was also managed with cyclophosphamide, vincristine and steroids (COP) protocol in view of multiple organ dysfunction at diagnosis requiring significant cardiorespiratory support. Subsequently with clinical stabilisation and histological confirmation, he was started on COG ALCL induction protocol with vincristine, prednisolone, doxorubicin and intrathecal methotrexate.

The PET scan conducted after induction chemotherapy showed complete metabolic response and he was continued on the consolidation phase. However, he had very early disease relapse in the left cervical lymphnodes immediately after the cycle 2 consolidation phase. After histological confirmation, he was started on salvage protocol with brentuximab vedotin, and bendamustine; however, he had only a partial response (Deauville score of 4 on PET-CT scan). He achieved remission (CR2) after six weeks of vinblastine and crizotinib based therapy and then underwent 10/10 matched unrelated donor hematopoietic stem cell transplant (HSCT) with fludarabine, melphalan, 4Gy total body irradiation-based conditioning. He has been started on crizotinib maintenance since D+60 post HSCT and is doing well six months post HSCT with complete chimerism.

## Discussion

Hemophagocytic lymphohistiocytosis is a life-threatening disorder caused by hyperinflammation or immune dysregulation, and is characterized by persistent fever, hepatosplenomegaly, and cytopenia. It can be classified into primary HLH due to genetic defects of perforin, Munc and syntaxin genes; or, secondary HLH associated with infections, rheumatological or autoimmune disorders and malignancies. Common malignancies associated with secondary HLH include leukemia (T/NK cell types), lymphomas, and Hodgkin’s lymphoma [3,4].

Anaplastic large cell lymphoma is a type of non-Hodgkin’s lymphoma (NHL) and constitutes 10% to 15% of paediatric NHL. The classical presentation of ALCL includes lymphadenopathy and fever with constitutional symptoms; extranodal manifestations including involvement of skin, lungs and bones are common in ALCL [5]. The constitutive expression and activation of oncogenic ALK fusion proteins, due to rearrangements of the ALK gene with the nucleophosmin gene (NPM1) leading to t (2:5) translocation, is seen in the majority of ALCL cases or, less frequently, with gene partners other than NPM1 [6].

 In a study by Han et al., the incidence of M-HLH was noted to be 1.9% among the patients with leukemia and lymphomas. The same study reported eight pediatric patients with M-HLH including two children with ALCL. No underlying genetic mutation was identified in these patients and one of them succumbed to the disease [7].

The activation of the immune system by a massive liberation of cytokines especially IL4, IL13 and TNFa, and an over expression of IL22R1 on the lymphocytes has also been described as a mechanism for the inflammatory state in ALCL, most of whom had HLH [8]. Anaplastic large cell lymphoma has been reported as one of the most common paediatric lymphomas to be associated with M-HLH or even leukemoid reaction, which often masks the underlying malignancy, hence delaying the diagnosis [9,10].

Presentation similar to sepsis or inflammatory disorder with extreme neutrophilic leucocytosis have been reported by Satish et al. reported in a three-year-old boy with pyrexia of unknown origin for three weeks, with no response to multiple courses of antibiotics. Examination revealed pallor, icterus, cervical and axillary lymphadenopathy, hepatosplenomegaly and polyserositis (characterized by ascites and bilateral pleural effusion). There were no classical features of HLH noticed. Evaluation with lymph node excision biopsy revealed ALCL and the patient improved with chemotherapy [11].

In a study on 50 paediatric patients with ALCL by Pasqualini et al. over a 24-year study period, 12% had M-HLH. The study reported complete remission in all these patients with induction therapy with two children having subsequent relapse. There was no significant difference in terms of age, gender and survival rates between the groups of children with or without HLH. Lung involvement with ALCL was more frequently reported in those with HLH in the study, as was seen in our patient [4].

In contrast, there are reports to suggest that ALCL associated with HLH have been described to be aggressive and is expected to have a resistance to chemotherapy. Our patient had a very early relapse and had subsequent refractory course prior to HSCT. However, with allogenic HSCT and crizotinib, he is doing well [12].

Compared to B cell NHLs and HLs, T cell NHLs have been reported to be frequently seen in males more than females and has been complicated with HLH and confer a worse prognosis [13]. Treatment options include intensive multiagent chemotherapy. The newer treatment options such as monoclonal antibodies including brentuximab vedotin, targeted agents like crizotinib against ALK protein and vinblastine monotherapy have changed the paradigm of management of relapsed refractory ALCL patients. Allogenic HSCT is reserved for those with refractory disease or early relapses and offers 50% to 60% successful outcomes [14].

## Conclusions

In children with prolonged fever with features of hyperinflammatory state like HLH, both infectious and noninfectious etiologies for HLH must be considered and must have a high index of suspicion for malignancy. It is important that pediatric hemato-oncologists be aware that M-HLH can mimic infection as in our patient who had a high spiking fever, consolidation, and mediastinal adenopathy. Therefore, a workup should be done for malignancy-associated complication when an infectious etiology is not identified after thorough evaluation.
